# Multiple coronary artery perforation as a fatal complication during the management of an undeflatable stent balloon: a case report

**DOI:** 10.3389/fcvm.2025.1565014

**Published:** 2025-03-17

**Authors:** Seok Hyun Kim, Sang Hyun Lee, Jeongsu Kim, Kook Jin Chun

**Affiliations:** Division of Cardiology, Department of Internal Medicine and Research Institute for Convergence of Biomedical Science and Technology, Pusan National University Yangsan Hospital, Pusan National University School of Medicine, Yangsan, Republic of Korea

**Keywords:** percutaneous coronary intervention, coronary occlusion, coronary device entrapment, complications, perforation

## Abstract

**Background:**

An undeflatable stent balloon following its inflation during percutaneous coronary intervention (PCI) is a rare and unpredictable complication that can lead to serious consequences. Currently, there is no standardized protocol for managing this issue.

**Case presentation:**

An 83-year-old man presented with chest pain. Coronary angiography showed a chronic total occlusion (CTO)-like lesion in the proximal left anterior descending coronary artery (LAD). Following stent deployment, the balloon failed to deflate and remained inflated within the LAD. Despite multiple retrieval attempts, the issue remained unresolved. As an alternative to surgical removal, we inflated the balloon beyond its rated burst pressure within the coronary artery. The balloon eventually ruptured and was successfully retrieved; However, this resulted in multiple severe coronary perforations, which were effectively sealed using covered stents.

**Conclusion:**

Balloon deflation failure is an exceptionally rare, unpredictable, and critical complication of PCI. While various troubleshooting strategies exist, inflating an undeflatable balloon beyond its burst pressure should be considered only as a last resort, with thorough preparation for potential complications.

## Background

Percutaneous coronary intervention (PCI) is a common therapeutic approach for treating coronary artery disease. Even the simplest interventional procedures can result in complications due to hardware failures, which are sometimes very challenging to manage. We report a rare complication of an undeflatable stent balloon during a PCI procedure, which led to a nearly fatal complication following the bailout approach.

## Case presentation

An 83-year-old man presented with an acute non-ST-segment elevation myocardial infarction was admitted to our hospital. His medical history included hypertension, diabetes, dyslipidemia, and stage 3 chronic kidney disease.

The initial ECG showed no ST elevation or diffuse ST depression, but poor R-progression was noted. CK-MB was elevated to 9.7 ng/ml, and high-sensitivity Troponin I to 130 pg/ml. A chest x-ray revealed mild pulmonary edema with an SpO₂ 91%–93%. Echocardiography detected new akinesis in the LAD territory.

**Figure 1:**
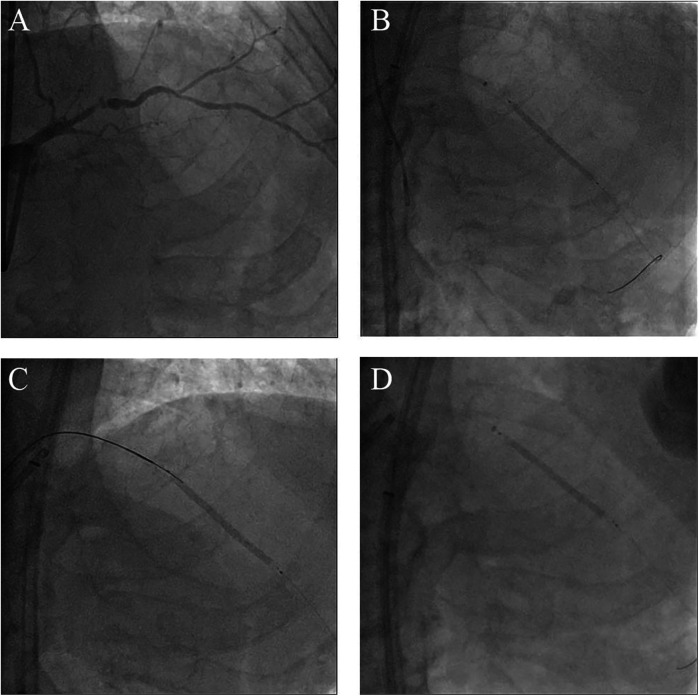
Morphology of the coronary lesions and several attempts to manage the undeflatable coronary balloon. **(A)** Coronary angiography from right anterior oblique cranial projection showing a CTO-like lesion of the proximal LAD. **(B)** Failure to deflate the stent balloon despite strong negative pressures using a saline-filled indeflator. **(C)** Attempt to perforate the undeflatable balloon using a stiff wire with microcatheter support. **(D)** Attempt to pull the undeflatable balloon back after deep intubation of the guide extension catheter; CTO, chronic total occlusion; LAD, left anterior descending artery.

**Figure 2:**
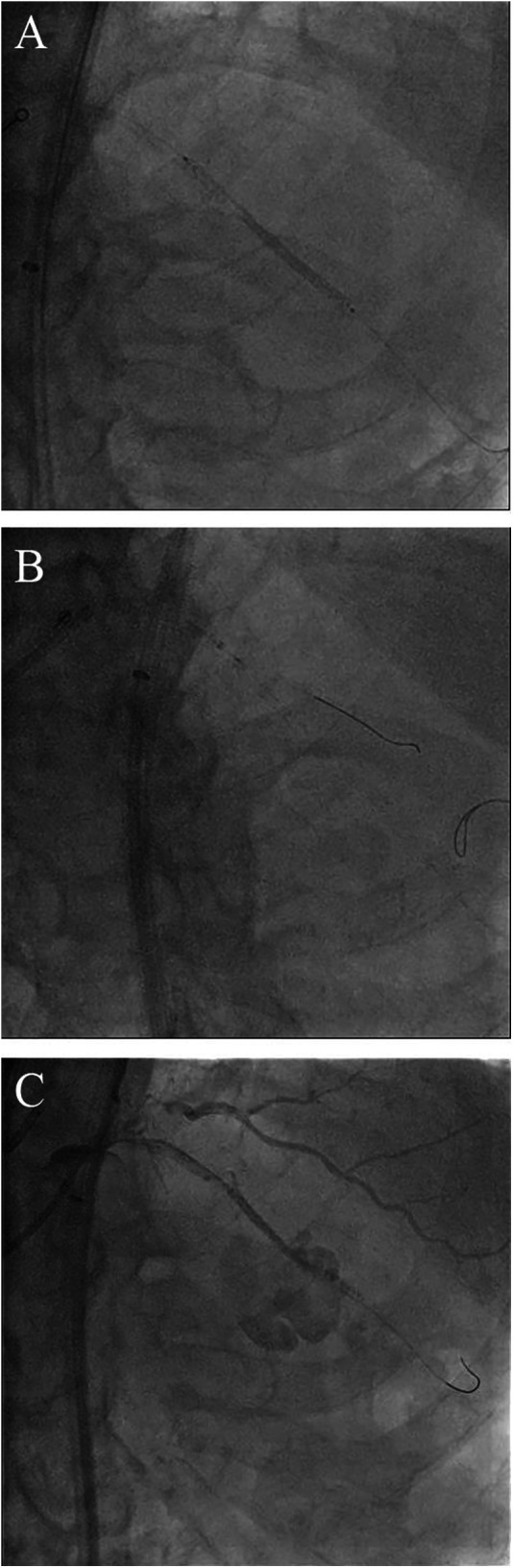
Bursting the balloon in the coronary artery with beyond-high-pressure inflation and subsequent coronary perforation. **(A)** Partial loss of contrast within the stent balloon following its rupture. **(B)** Complete removal of the ruptured stent balloon into the guiding catheter. **(C)** Large coronary artery perforation after the balloon burst.

**Figure 3:**
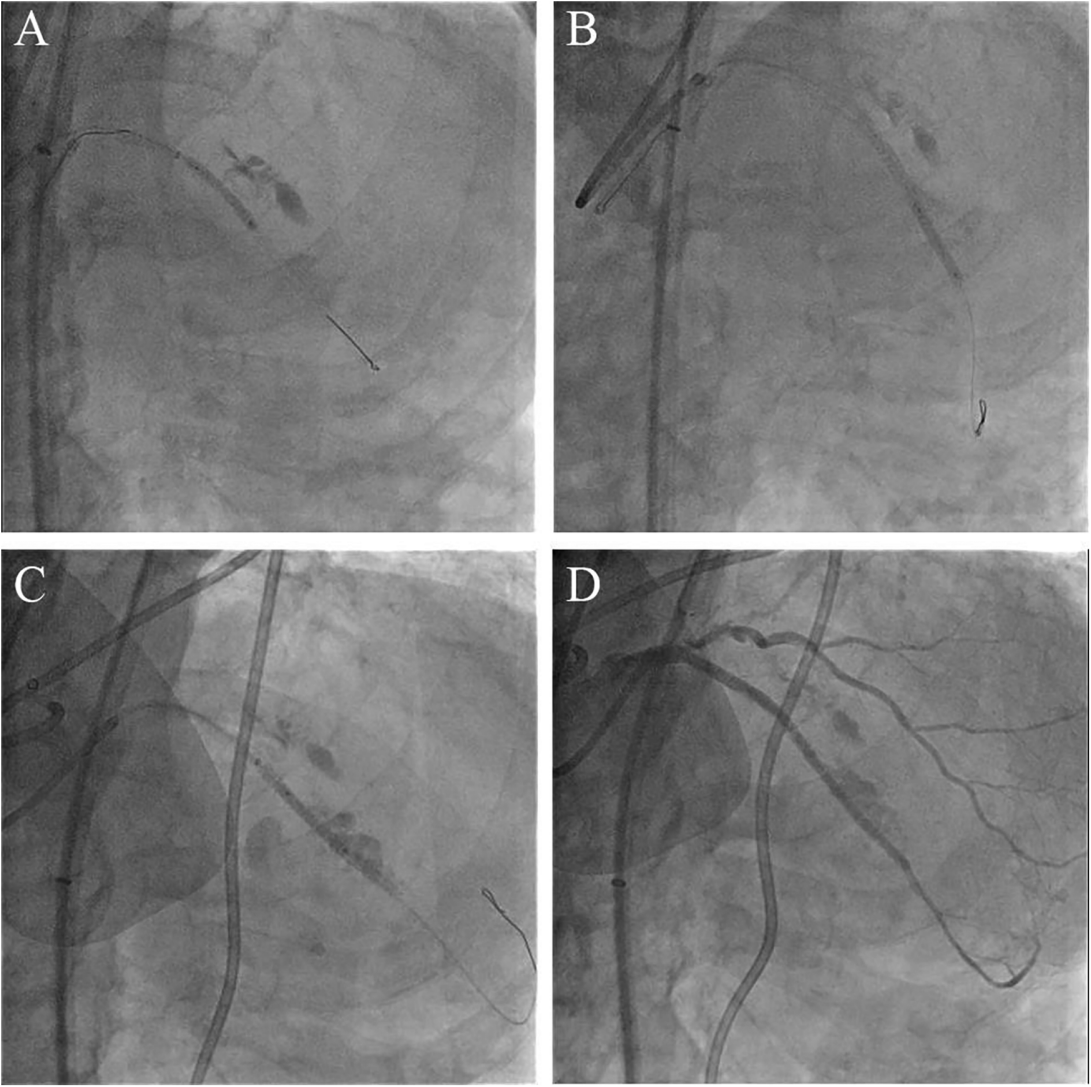
Salvage procedure for coronary perforation employing the ping-pong technique. **(A)** The covered stent deployed at the proximal segment of stented LAD. **(B)** The second covered stent placed in the distal segment of stented LAD. **(C)** Final perforation closure with a third covered stent. **(D)** Angiogram after deploying three covered stents shows complete sealing of the perforation.

Coronary angiography, performed as part of an early invasive strategy, revealed a severely calcified chronic total occlusion (CTO)-like lesion in the proximal LAD and calcified stenosis of the ramus intermedius branch (Figure 1A). Right coronary angiography showed no significant stenosis, with CC grade 1 collaterals supplying the LAD via the epicardial and septal vessels. During two days of diuresis and stabilization, the patient's chest pain and dyspnea improved, and no dynamic ST-segment changes were observed. On day 3, PCI was performed according to the patient's preference. The laboratory findings on the day of PCI showed CK-MB 82 ng/ml and high-sensitivity Troponin I 13,138 pg/ml. Both common femoral arteries were accessed for PCI. An 8 F EBU 3.5 guiding catheter (Medtronic, Minneapolis, MN, USA) was used to engage the left main ostium. The LAD was successfully wired using the antegrade wire escalation technique, a commonly used approach in contemporary CTO PCI. (Escalation from Sion BLUE (Asahi Intecc, Japan) → Fielder XT-R (Asahi Intecc, Japan) → Ultimate Bros 3.0 (Asahi Intecc, Japan), with Corsair Pro XS (Asahi Intecc, Japan) microcatheter back-up.) 7F Guidezilla™ extension catheter (Boston Scientific, Natick, MA, USA) was introduced, and lesion preparation was sequentially performed using a 2.0 mm semi-compliant balloon followed by a 2.5 mm non-compliant balloon. Intravascular ultrasound confirmed the wire was in the true lumen throughout the LAD, revealing diffuse and calcified stenosis from the proximal to the distal LAD. Subsequently, a 2.5 × 46 mm Cre8™ EVO (Alvimedica, Istanbul, Turkey) drug-eluting stent (DES) was delivered and positioned across the mid-LAD lesion without resistance. During stent deployment, the dial on the pressure gauge window did not rise appropriately despite multiple adjustments to the indeflator handle. After approximately 15 attempts, we reached the rated burst pressure of 18 atm, achieving full stent balloon expansion (Figure 1B). However, retraction of the handle failed to deflate the balloon. Despite the dial indicating zero pressure and a strong tactile sensation of negative pressure when pulling back the handle, the stent balloon remained inflated with no signs of deflation. Our initial suspicion was a malfunctioning indeflator device. Therefore, we immediately switched to a new indeflator, filling the column with normal saline alone to dilute the contrast/saline mixture in the balloon catheter through slight inflation followed by full deflation. However, this also failed to deflate the balloon. We then attached a three-way stopcock connected to a 50 cc syringe to the balloon catheter and applied strong negative suction, but this also proved unsuccessful. After all efforts to deflate the balloon using negative pressure failed, we decided to attempt intentional balloon perforation using a stiff guidewire. A 7 F Judkins Left 4 (JL 4) guide catheter was inserted via the left femoral artery. The first EBU guide catheter was slightly withdrawn, and the JL 4 guide catheter was advanced into the left main. A Turnpike® LP microcatheter (Teleflex, Wayne, PA, USA) was used to deliver the wire. We attempted to puncture the inflated balloon with both the distal and proximal end of a Conquest Pro 12 and Astato® XS 20 wire (Asahi Intecc, Japan) multiple times (Figure 1C), but these attempts were unsuccessful. As the next step, we advanced the GuideZilla™ guide extension catheter deeply and attempted forceful retraction of the trapped stent balloon, which again failed (Figure 1D). Meanwhile, the patient developed severe chest pain and ST elevation on the electrocardiogram monitoring. His hemodynamic status deteriorated, necessitating the initiation of norepinephrine and dopamine. We contacted the cardiothoracic surgeon to discuss surgical options. To avoid delays in this critical patient, we decided to attempt ultra-high-pressure inflation of the balloon beyond its rated burst pressure as a last interventional resort. At 23 atm, the pressure dial suddenly dropped, and the contrast dissipated from the balloon, indicating a rupture (Figure 2A). The balloon was then successfully retrieved into the guiding catheter and removed from the coronary artery (Figure 2B). However, follow-up angiography revealed multiple Ellis grade III perforations in the stented LAD segment (Figure 2C). A 2.5 mm semi-compliant balloon was immediately inflated in the mid-LAD to plug the perforation. Despite prolonged inflation for 15 min, the perforation remained unsealed, necessitating the deployment of 3.5 × 19 and 2.8 × 19 mm GraftMaster covered stents (Abbott Vascular, Santa Clara, CA, USA) in the proximal and distal segments of the stented LAD using the Ping Pong technique (Figures 3A,B). As the patient's hemodynamic status remained unstable, an emergency pericardiocentesis was performed. Angiography still showed persistent extravasation in the mid-segment of the stented LAD, so an additional 2.8 × 19 mm GraftMaster covered stent was deployed (Figure 3C). After successfully managing the coronary perforation, a 3.0 × 33 mm DES was placed in the stenotic proximal LAD, followed by post-dilation with a 3.5 mm non-compliant balloon. Final angiography confirmed well-expanded stents with no dissection or residual perforation (Figure 3D). Fortunately, the patient was discharged after 19 days (including three days in the intensive care unit) and remained asymptomatic for two months. We initially planned to place him on lifelong dual antiplatelet therapy, but he was later transferred to an elderly care hospital and lost to follow-up.

## Discussion

Deflation failure of a stent balloon during PCI is a very rare complication (1). Several mechanisms may contribute to this issue, including kinking, twisting, or stretching of the balloon catheter during delivery or lesion crossing, which can prevent proper deflation after inflation (2). Another potential causes include acute recoil of a heavily calcified lesion or balloon entrapment within the guide catheter (3). In our case, the difficulty was observed not only in deflating the balloon but also during initial inflation, suggesting the possibility of an unintentional kink in the balloon shaft or a manufacturing defect. When stent or balloon catheter delivery is particularly challenging, increasing the risk of hypotube compromise, a guide extension catheter may help facilitate the procedure. However, this was not applicable in our case. An inflated balloon within a coronary artery can completely block the vessel, leading to life-threatening complications such as ischemia, infarction, malignant arrhythmia, and death. Therefore, emergent rescue intervention is critical when a stent balloon fails to deflate, unlike entrapments of other interventional devices. Several techniques have been proposed in previous case reports to address an undeflatable stent balloon.

**Figure 4:**
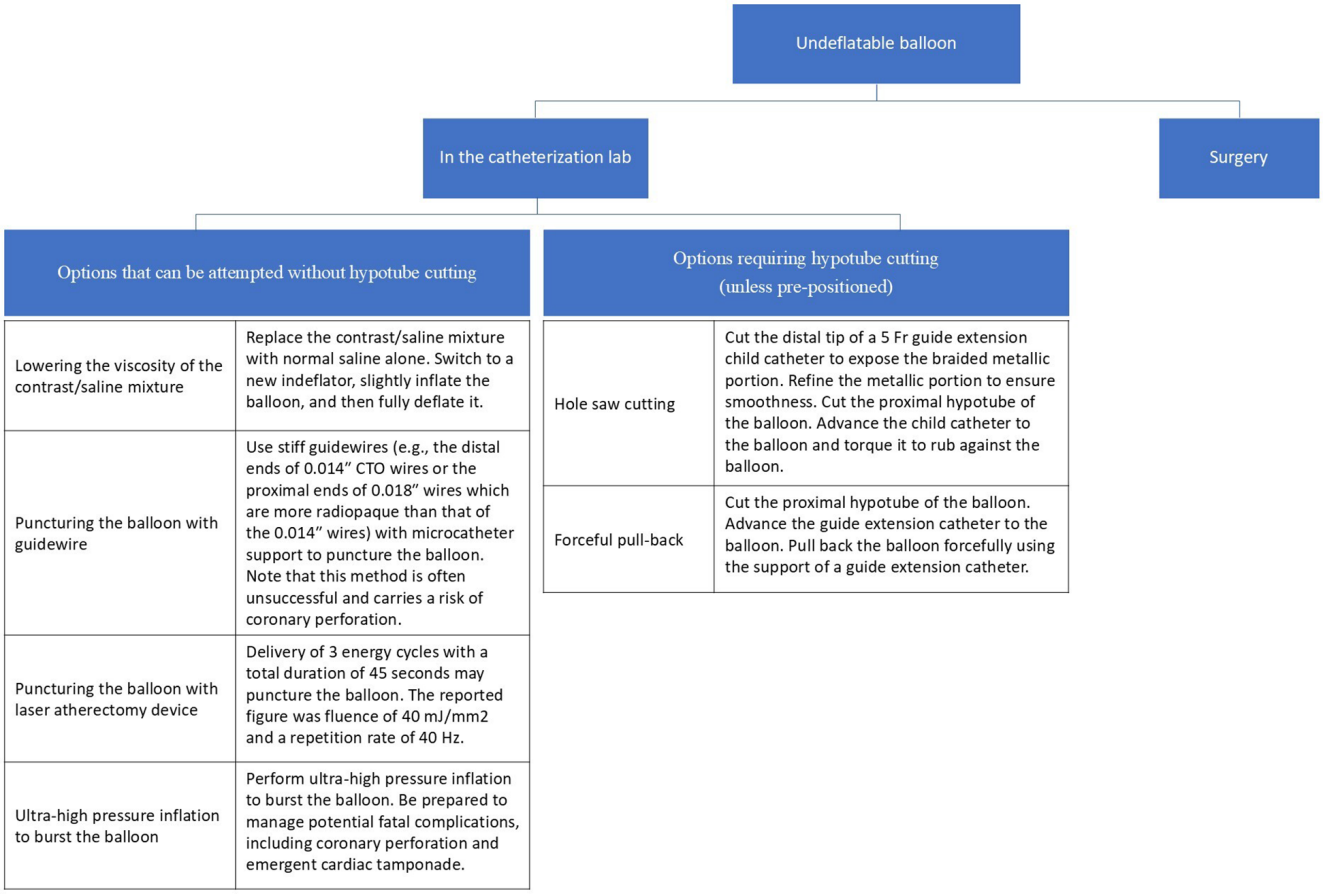
Suggested treatment options for an undeflatable balloon. The possible interventions in the catheterization room are broadly categorized into those that require hypotube cutting and those that do not.

The retrieval techniques available in the catheterization laboratory can be broadly classified into those that do not require cutting the hypotube and those that necessitate cutting the hypotube. While there is no universally established order, we have outlined the techniques in a structured sequence in Figure 4.

### Techniques without cutting the hypotube

First-line: Contrast dilution and negative pressure techniques.

The first-line approach involves progressive dilution of the contrast material within the balloon using saline to decrease viscosity (2). Additionally, using dual indeflators connected via a stopcock can enhance suction power and facilitate balloon deflation.

Second-line: Guidewire puncture.

The distal end of 0.014″ CTO wires or the proximal end of 0.018″ wires (which are more radiopaque) can be used to puncture the balloon with microcatheter support, although this method is rarely successful (2, 4).

Third-line: Laser energy delivery.

Successful balloon deflation has been reported following laser energy delivery at 40 mJ/mm^2^ fluence and 40 Hz repetition rate, applied in three cycles for a total duration of 45 s (5).

Fourth-line: Ultra-high-pressure inflation.

As a final option before cutting the hypotube, the balloon can be intentionally ruptured using ultra-high-pressure inflation. However, this carries a significant risk of coronary artery perforation and should be avoided unless absolutely necessary (1–4, 6, 7).

### Techniques that require cutting the hypotube

In some cases, cutting the hypotube does not guarantee passive balloon deflation and eliminates the option of using an indeflator. However, if necessary, the following methods can be attempted:

First-line: Hole saw technique using a child guide catheter.

Cutting the distal tip of a child guide catheter (e.g., 5 Fr Terumo Heartrail) exposes its braided metallic skeleton, which can then be used to mechanically perforate the balloon by rubbing against it (6).

Second-line: Forceful retraction with guide-extension catheter support.

Forceful retraction of the balloon using guide-extension support can be attempted. However, this method carries a risk of a deeply intubated guide catheter with hypotube disruption (3, 6, 7).

### Last-resort option

If all catheter-based retrieval attempts fail, surgical balloon retrieval remains a definitive option. However, due to its invasiveness and procedural delays, an interventional approach should be prioritized unless there is ongoing hemodynamic compromise (8).

Although we successfully managed the undeflatable stent balloon by inflating it beyond high pressure, this approach resulted in multiple serious coronary artery perforations. To our knowledge, this salvage procedure has never been reported as a successful intervention inside a coronary artery. Nevertheless, alternative strategies should always be considered before employing this drastic ultra-high pressure balloon bursting method.

The mechanism underlying edge perforation may be attributable to high intramural stress. Overexpanding a stent is functionally equivalent to implanting an oversized stent, which increases intramural stress, particularly at the stent edges (9). However, in this case, a mid-segment perforation necessitated the placement of an additional covered stent. Another potential cause of mid-stent perforation is pinhole balloon rupture. A semi-compliant stent balloon generally expands to approximately 1.3 times its nominal diameter at rated burst pressure. However, specific burst pressures and pressure-burst relationships are often unspecified. Studies have shown that second-generation DES can expand beyond 50% of their nominal diameter during post-dilation with a larger balloon, though the extent of expansion varies depending on the stent design. Therefore, in our case, the mid-stented segment perforation may not have resulted solely from overdistension of the stent but could also be attributed to pinhole perforation of the balloon, as previously documented, particularly in heavily calcified lesions (10, 11). Deploying a long covered stent in the LAD inherently compromises blood flow to multiple critical branches, which can lead to a large infarct territory. However, in this patient, the presence of CTO-like chronic ischemia, compounded by acute ischemia but partially compensated by collateral circulation, helped mitigate the impact of branch occlusion. Given these risks, this approach should only be attempted as an last resort, with comprehensive preparation for potential perforation-related complications. A summary of troubleshooting techniques for an undeflatable stent balloon, along with additional case resources, is available online (12).

## Conclusion

Failure to deflate a coronary balloon catheter is an extremely rare but potentially life-threatening complication during PCI. In our case, conventional retrieval techniques were unsuccessful, ultimately necessitating balloon rupture via ultra-high-pressure inflation as a last resort. While this approach successfully removed the balloon, it resulted in multiple coronary perforations requiring extensive intervention.

To our knowledge, this is the first reported case of a successful stent balloon retrieval using this extreme method within a coronary artery. However, given its high risk, alternative strategies should always be prioritized before considering ultra-high-pressure inflation.

This case highlights the importance of preparing for rare but severe complications during complex PCI procedures. Bursting an undeflatable balloon inside a coronary artery should only be attempted in exceptional circumstances, with thorough preparation for potential catastrophic events, including coronary perforation management.

## Data Availability

The original contributions presented in the study are included in the article further inquiries can be directed to the corresponding author.
